# Prognostic N6-methyladenosine (m6A)-related lncRNA patterns to aid therapy in pancreatic ductal adenocarcinoma

**DOI:** 10.3389/fgene.2022.866340

**Published:** 2022-09-26

**Authors:** Yuxin Wang, Yutian Ji, Qianhui Xu, Wen Huang

**Affiliations:** ^1^ Department of Orthopaedic Surgery, The First Affiliated Hospital, Zhejiang University School of Medicine, Hangzhou, China; ^2^ School of Medicine, Zhejiang University, Hangzhou, Zhejiang, China; ^3^ The Second Affiliated Hospital and Yuying Children’s Hospital of Wenzhou Medical University, Wenzhou, Zhejiang, China

**Keywords:** pancreatic ductal adenocarcinoma, m6A-related lncRNA patterns, tumor microenvironment, prognostic prediction, molecular mechanism

## Abstract

**Background:** Mounting research studies have suggested the indispensable roles of N6-methyladenosine (m6A) RNA modification in carcinogenesis. Nevertheless, it was little known about the potential function of m6A-related lncRNAs in sample clustering, underlying mechanism, and anticancer immunity of pancreatic ductal adenocarcinoma (PDAC).

**Methods:** PDAC sample data were obtained from TCGA-PAAD project, and a total of 23 m6A regulators were employed based on published articles. Pearson correlation and univariate Cox regression were analyzed to determine m6A-related lncRNAs with prognostic significance to identify distinct m6A-related lncRNA subtypes by consensus clustering. Next, the least absolute shrinkage and selection operator (LASSO) algorithm was applied for constructing an m6A-related lncRNA scoring system, further quantifying the m6A-related lncRNA patterns in individual samples. Gene set variation analysis (GSVA) was employed to assign pathway activity estimates to individual samples. To decode the comprehensive landscape of TME, the CIBERSORT method and ESTIMATE algorithm were analyzed. The half-maximal inhibitory concentration (IC_50_) of chemotherapeutic agents was predicted with the R package pRRophetic. Finally, a quantitative real-time polymerase chain reaction was used to determine TRPC7-AS1 mRNA expression in PDAC.

**Results:** Two distinct m6A-related lncRNA patterns with different clinical outcomes, TEM features, and biological enrichment were identified based on 45 prognostic m6A-related lncRNAs. The identification of m6A-related lncRNA patterns within individual samples based on risk scores contributed to revealing biological signatures, clinical outcomes, TEM characterization, and chemotherapeutic effects. A prognostic risk-clinical nomogram was constructed and confirmed to estimate m6A-related lncRNA patterns in individual samples. Finally, the biological roles of TRPC7-AS1 were revealed in PDAC.

**Conclusion:** This work comprehensively elucidated that m6A-related lncRNA patterns served as an indispensable player in prognostic prediction and TEM features. Quantitative identification of m6A-related lncRNA patterns in individual tumors will contribute to sample stratification for further optimizing therapeutic strategies.

## Introduction

Methylation of N6 adenosine (m6A), characterized as the most predominant type of RNA modification, serves as a pivotal regulator in multiple biological progression and pathological processes (Zhao et al., 2017; He et al., 2019). m6A modification was mainly controlled by dynamic and reversible regulation of methylation enzymes identified as the binding proteins (readers), the demethylases (erasers), and methyltransferases (writers) (Zaccara et al., 2019). Furthermore, m6A modification was affected by the expression pattern and biological function of these methylase complexes, and the exploration of these regulatory proteins contributes to the determination of underlying mechanisms of m6A modification (Zhou et al., 2020). Accumulating studies have supported that abnormal expression and mutation variation of m6A regulators held crucial players in tumorigenicity and dysregulated immunity (Chen and Wong, 2020; Shulman and Stern-Ginossar, 2020). A comprehensive landscape of the expression pattern and genetic alteration of m6A will facilitate the recognition of m6A-based therapeutic targets further predict prognosis and improve clinical outcomes accordingly (Li et al., 2019).

Pancreatic ductal adenocarcinoma (PDAC) is one of the most common human cancers and the seventh leading reason of tumor-associated death globally (Mizrahi et al., 2020). There were approximately 496,000 newly diagnosed patients and almost 466,000 related deaths according to the 2020 global cancer statistics (Sung et al., 2021). Given the difficulty of early precision diagnosis and rapid tumor progression, a large number of PDAC cases presented advanced clinical stage or distant metastatic disease at diagnosis (Mizrahi et al., 2020; Tang et al., 2021). It is of great importance, thus, to develop novel and reliable indicators for prognostic estimation and therapeutic efficacy prediction to further advance tailored therapy.

Currently, antitumor immunotherapy has attracted people’s interest with the flourish of immune checkpoint inhibitors, but only a minority of cancer patients could benefit from them. Immune checkpoint blockade immunotherapy (anti-CTLA-4) has made great progress in numerous malignant cancers; however, the results of clinical trials remained unsatisfactory in PDAC (Royal et al., 2010; Brahmer et al., 2012). The immunosuppressive tumor microenvironment (TEM) contributed to limited therapeutic effect of immunotherapy (O'Reilly et al., 2019). Account for almost half of immune infiltration and tumor cellular population functioned as opposing players in anticancer immunity (Clark et al., 2007). There are increasing numbers of tumor-associated fibroblasts, regulatory T cells, myeloid-derived suppressor cells, and tumor-associated macrophages in TEM, most of which significantly inhibited antitumor immunotherapy (Hessmann et al., 2020).

Long noncoding RNA (lncRNA) with >200 bp RNA transcripts did not possess the ability of protein-coding (Wang et al., 2011). Currently, an increasing number of studies have suggested that lncRNAs have critical roles in regulation of anticancer immunity, including immune activation and antigen release (Carpenter and Fitzgerald, 2018; Denaro et al., 2019). Notably, more and more researchers concluded that m6A and lncRNAs may share synergistic interactions in cancer progression (Ma et al., 2019).

An independent research study indicated that ALKBH5 was discovered to cooperate with lncRNA forkhead box protein M1 (FOXM1)-AS to accelerate tumorigenicity and proliferation of glioblastoma stem cells (GSCs) (Zhang et al., 2017). Moreover, the m6A modification level of lncRNA 1281 could significantly regulate the differentiation of embryonic stem cells (ESCs) by affecting let-7 levels (Yang et al., 2018). A previous research study has proposed the m6A-related lncRNA risk model for predicting prognosis for patients with pancreatic adenocarcinoma. Nevertheless, m6A-related lncRNAs involved in subtype identification, underlying mechanism, and chemotherapeutic prediction in PDAC remained to be revealed.

In this work, m6A-related lncRNA patterns were comprehensively analyzed by using the transcriptomic information of PDAC samples from TCGA-PAAD project. Two different m6A-related lncRNA pattern subtypes were identified using consensus clustering, and biological processes of different clusters were assigned. In addition, an m6A-related lncRNA scoring scheme was constructed to quantify the m6A-related lncRNA risk of each sample. Finally, the underlying signaling pathways, TEM features, and chemotherapeutic prediction were analyzed under the risk score. Finally, the biological functions of TRPC7-AS1 in prognostic prediction and pathway enrichment were further explored to provide robust insights into the clinical therapeutic strategy in PDAC. Our findings highlighted that m6A-related lncRNAs played critical roles in prognostic prediction and tumor progression in PDAC, facilitating advanced therapeutic strategies.

## Methods and materials

### Public dataset collection and preprocessing

Gene-expression annotation and clinical information were obtained from The Cancer Genome Atlas (TCGA, https://cancergenome.nih.gov/) database. In total, 177 PDAC samples from TCGA-PAAD project were used for comprehensive analysis. TCGA RNA sequencing information (FPKM format) of gene expression was obtained from the Genomic Data Commons (GDC, https://portal.gdc.cancer.gov/) and transformed into transcripts per kilobase million (TPM) value. The genomic mutation profiles including simple nucleotide variation (SNV) and copy number variation (CNV) of TCGA-PAAD cohort were curated from the Genomic Data Commons (GDC, https://portal.gdc.cancer.gov/). The copy number variation of 23 m6A regulators was plotted using the “Rcircos” R package in human chromosomes. The analysis process flow chart is presented in Supplementary Figure S1.

### Identification of prognostic m6A-related lncRNAs

The lncRNA information was identified using a constructed mining method with reference to Xu et al. (2021a). Briefly, genes were recognized as non-coding genes or protein-coding genes according to their Refseq IDs or Ensembl IDs, and only the long non-coding genes in NetAffx annotation files were retained. According to existing research studies focusing on m6A modification, a total of 23 acknowledged m6A methylation modification regulators were gathered and analyzed to uncover m6A methylation modification patterns (Zhao et al., 2017; Chen et al., 2019; He et al., 2019; Zaccara et al., 2019). These m6A regulators constitute 13 readers (ELAVL1, FMR1, HNRNPA2B1, HNRNPC, IGFBP1, IGFBP2, IGFBP3, LRPPRC, YTHDC1, YTHDC2, YTHDF1, YTHDF2, and YTHDF3), 8 writers (CBLL1, KIAA1429, METTL14, METTL3, RBM15, RBM15B, WTAP, and ZC3H13), and 2 erasers (ALKBH5 and FTO). The expression levels of 23 m6A regulators of the TCGA-PAAD project are detected and listed in Supplementary Table S1. Pearson correlation was analyzed to explore the correlation of m6A regulators with lncRNAs. The lncRNAs with correlation coefficient |R| > 0.4 and *p* < 0.001 was considered as m6A-related lncRNAs. Next, univariate cox regression analysis within R package “survival” was performed to determine prognostic m6A-related lncRNAs (*p* < 0.01).

### Consensus clustering based on prognostic m6A-related lncRNAs

Unsupervised clustering analysis was conducted to determine distinct m6A-related lncRNAs patterns based on the gene-expression data of prognostic m6A-related lncRNAs for stratification of samples for further analysis. The number of clusters was identified by the consensus clustering algorithm based on their stability. R package “ConsensusClusterPlus” was used to implement these analyses, and repetitions of 1,000 times were performed for guaranteeing the stability of clustering.

### Gene set variation analysis

GSVA analysis [40] with the ‘GSVA’ R package was used to explore the variation in biological processes between distinct m6A-related lncRNAs patterns. The well-defined biological signatures were derived from the gene sets of “c2. cp.kegg.v7.4. symbols.gmt” and “h.all.v7.4. symbols.gmt” (downloaded from the Molecular Signatures Database).

### Characterization of tumor microenvironment

The deconvolution approach CIBERSORT (http://cibersort.stanford.edu/) was used to estimate the abundances of 22 distinct leukocyte subsets with the gene expression profile. The Estimation of Stromal and Immune Cells in Malignant Tumors using Expression Data (ESTIMATE) algorithm (Yoshihara et al., 2013), as a new algorithm based on the unique properties of the transcriptional profiles, could estimate the tumor cellularity and the tumor purity. The immune score and stromal score were calculated to quantify the relative enrichment of immune and stromal cells, which form the basis for the ESTIMATE score to predict tumor purity.

### Construction of an m6A-related lncRNA prognostic signature

In total, 177 PDAC samples were randomly classified into the training and testing group with the rate of 3:2 using the R project “caret” package. Both training set and validation set needed to comply with the following requirements: 1) cases were stochastically classified as the training group and testing group; 2) samples in the two groups had similar clinicopathological traits. The testing cohort with 69 samples was further used to validate results derived from the training group. The LASSO regression was analyzed to eliminate the highly correlated m6A-related lncRNAs with the “glmnet” R package. The independent variable in the regression was the normalized expression matrix of candidate prognostic m6A-related lncRNAs, and the response variables were the overall survival and survival status of samples. The risk scores of each sample were calculated based on the expression level of each m6A-related lncRNA and its corresponding regression coefficients. The formula was established as follows: score = esum (each gene’s expression × corresponding coefficient). Finally, a risk model consisting of 11 prognostic m6A-related lncRNAs was established, and the risk score is computed using the formula: Risk score = βlncRNA 1 ×expression level of lncRNA 1 +βlncRNA 2×expression level of lncRNA 2 + · ···· +βlncRNA n × expression level of lncRNA n. Here, β was the regression coefficient in the LASSO Cox regression analysis. PDAC samples were assigned into low-/high-risk subgroups after setting the median value of risk score as the cut-off point**.**

### Validation of the m6A-related lncRNA prognostic signature

First, K–M survival curves were analyzed using the R package “survival”. Then, the receiver operating characteristic (ROC) curves were used to assess the prognostic significance. Subsequently, univariate and multivariate Cox regression were analyzed for independent validity of the risk score.

### Risk score with clinicopathological traits

To reveal the clinical value of risk score, correlation analysis of risk score with age, gender, tumor grade, pathological staging, and TNM categories was analyzed. R package “pheatmap” was used to visualize the distribution of clinicopathological variables in low-/high-risk groups.

### Depiction of the prognostic nomogram

To estimate the synergistic effect of risk score, age, gender, tumor grade, clinical stage, and TNM status in prognostic prediction for the overall survival rate, ROC curves were plotted to calculate the area under the curve (AUC) values (Blanche et al., 2013). To predict overall survival time in a quantitative manner, a prognostic nomogram including risk score and clinical variables was established to predict the 1/2/3-OS rate. Next, the calibration curve, which showed the prognostic value of the as-constructed nomogram, was constructed.

### Prediction of chemotherapeutic effect

To estimate the sensitivity of chemotherapy, the R package pRRophetic was used to estimate the half-maximal inhibitory concentration (IC_50_) of PDAC samples in different risk score groups. By constructing the ridge regression model based on the Genomics of Drug Sensitivity in Cancer (GDSC) (www.cancerrxgene.org/) cell line expression spectrum and TCGA gene expression profiles, the package pRRophetic could estimate IC_50_ of chemotherapeutic drugs (Geeleher et al., 2014).

### Experimental validation

HPNE (human pancreatic cell line) and three human pancreatic cancer cell lines (CFPAC-1 cells, PANC-1 cells, and SW1990 cells) were purchased from the Cell Bank of the Type Culture Collection of the Chinese Academy of Sciences, Shanghai Institute of Biochemistry and Cell Biology. The cell lines were all cultured in Roswell Park Memorial Institute (RPMI-1640) medium plus 10% fetal bovine serum (FBS; Invitrogen, Carlsbad, CA, USA). All cell lines were grown without antibiotics in a humidified atmosphere of 5% CO2 and 99% relative humidity at 37°C. Three different cell lines were subjected to a quantitative real-time polymerase chain reaction (qRT-PCR). Quantitative real-time PCR was analyzed as described previously (Xu et al., 2021b). All samples were analyzed in triplicates. Glyceraldehyde-3-phosphate dehydrogenase (GAPDH) levels were used as the endogenous control, and relative expression of TRPC7-AS1 was calculated using the 2^-ΔΔCt^ method. The sequences of primers used for PCR were as follows: TRPC7-AS1, 5′-GCC​TCC​TCC​TTC​CAT​AAC​G-3′ (forward) and 5′-CCC​ACA​GCC​TAG​ACC​CAT​T-3′ (reverse); and GAPDH, 5′-CAG​GAG​GCA​TTG​CTG​ATG​AT-3′ (forward) and 5′-GAA​GGC​TGG​GGC​TCA​TTT-3′ (reverse).

### Statistical analyses

The statistical analyses in this study were generated by R-4.0.3. For quantitative data, statistical significance for normally distributed variables was estimated using Student’s t-tests, and nonnormally distributed variables were analyzed using the Wilcoxon rank-sum test. For comparisons of more than two groups, Kruskal–Wallis tests and one-way analysis of variance were used as nonparametric and parametric methods, respectively. All comparisons were two-sided with an alpha level of 0.05, and the Benjamini–Hochberg method was applied to control the false discovery rate (FDR) for multiple hypothesis testing.

## Results

### Genetic variation of m6A RNA methylation regulators

**FIGURE 1:**
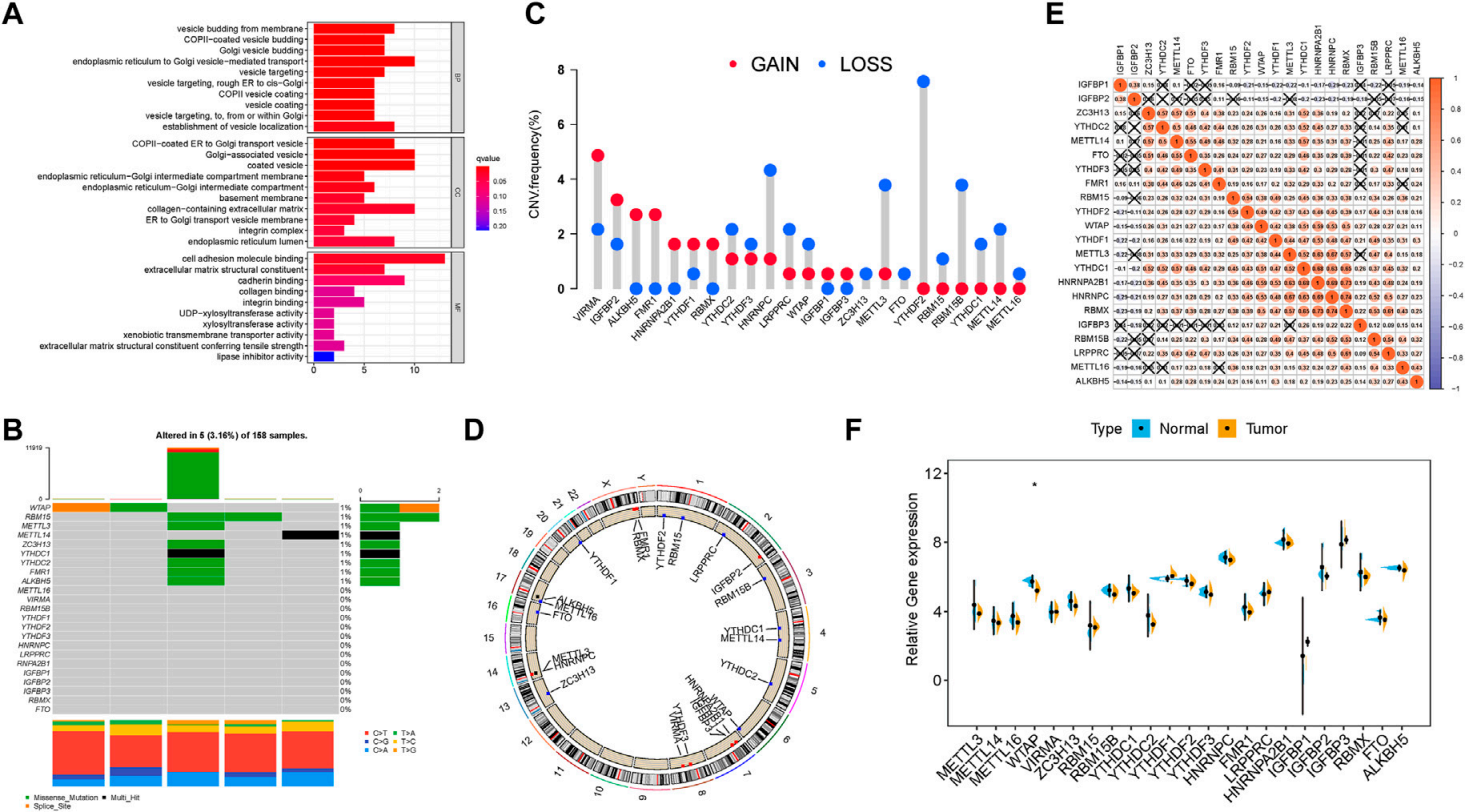
Landscape of genetic alterations of m6A regulators in PDAC. **(A)** GO enrichment analysis of the 23 m6A regulators. The *x*-axis indicates the gene ratio within each GO term. **(B)** Five of the 158 HCC patients experienced genetic alterations of 23 m6A regulators, with a frequency of 3.16%, mostly including amplification, missense mutations, and deep deletions. The number on the right indicates the mutation frequency in each regulator. Each column represents an individual patient. **(C)** CNV mutation frequency of 23 m6A regulators was prevalent. The column represents the alteration frequency. The deletion frequency is represented by the green dot; the amplification frequency is represented by the red dot. **(D)** Location of CNV alteration of m6A regulators on chromosomes. **(E)** Broad co-expression correlation among the 23 m6A RNA modification regulators in PDAC. “X” means *P* ≥ 0.05. **(F)** Difference in mRNA expression levels of 23 m6A regulators between normal and tumor samples. The asterisks represent the statistical *p*-value (*p < 0.05; **p < 0.01; ***p < 0.001).

In this research, the potential roles of 23 m6A modification regulators (“readers”: ELAVL1, FMR1, HNRNPA2B1, HNRNPC, IGFBP1, IGFBP2, IGFBP3, LRPPRC, YTHDC1, YTHDC2, YTHDF1, YTHDF2, and YTHDF3; “writers”: CBLL1, KIAA1429, METTL14, METTL3, RBM15, RBM15B, WTAP, and ZC3H13; and “erasers”: ALKBH5 and FTO) were explored in PDAC. GO annotation analyses of 23 m6A regulators were performed, and significant enrichment of biological pathways was visualized (Figure 1A). Subsequently, the landscape of mutation profiles of 23 m6A regulators in the TCGA-PAAD samples was delineated, from which we could discover that a total of 5 of 158 (3.16%) samples possessed somatic mutations of m6A regulators (Figure 1B). We exhibited that the top ten mutated genes in PDAC with ranked percentages, including WTAP (1%), RBM15 (1%), METTL3 (1%), METTL14 (1%), ZC3H13 (1%), YTHDC1 (1%), YTHDC2 (1%), FMR1 (1%), and ALKBH5 (1%). The prevalence of CNV mutations of 23 m6A regulators was further analyzed and presented that VIRMA, IGFBP2, ALKBH5, and FMR1 experienced prevalent CNV amplification, whereas YTHDF2, HNRNPC, METTL3, RBM15B, and METTL14 possessed widespread CNV deletions (Figure 1C). The chromosome locations of CNV mutations of these 23 m6A regulators are presented in Figure 1D. To elucidate mutual connection among 23 m6A regulators, Spearman correlation was analyzed (Figure 1E). The results showed that readers IGFBP1 and IGFBP2 presented a significant positive relation with other m6A regulators, while other 21 m6A regulators were positively correlated with each other. However, it was discovered that there was a nonsignificant difference in most m6A regulators’ expression levels between tumor and normal tissues (Figure 1F). Notably, the expression level of 13 m6A regulators could predict PDAC patients’ overall prognosis (Supplementary Figure S2). These results supported the significant distinctions and intrinsic interactions in the transcriptomic and genomic information of m6A RNA modification regulators in tumors of PDAC. Thus, the genetic variations of m6A RNA modification regulators might contribute novel insight into tumorigenicity and progression of PDAC.

### Identification of prognostic m6A-related lncRNAs

**FIGURE 2:**
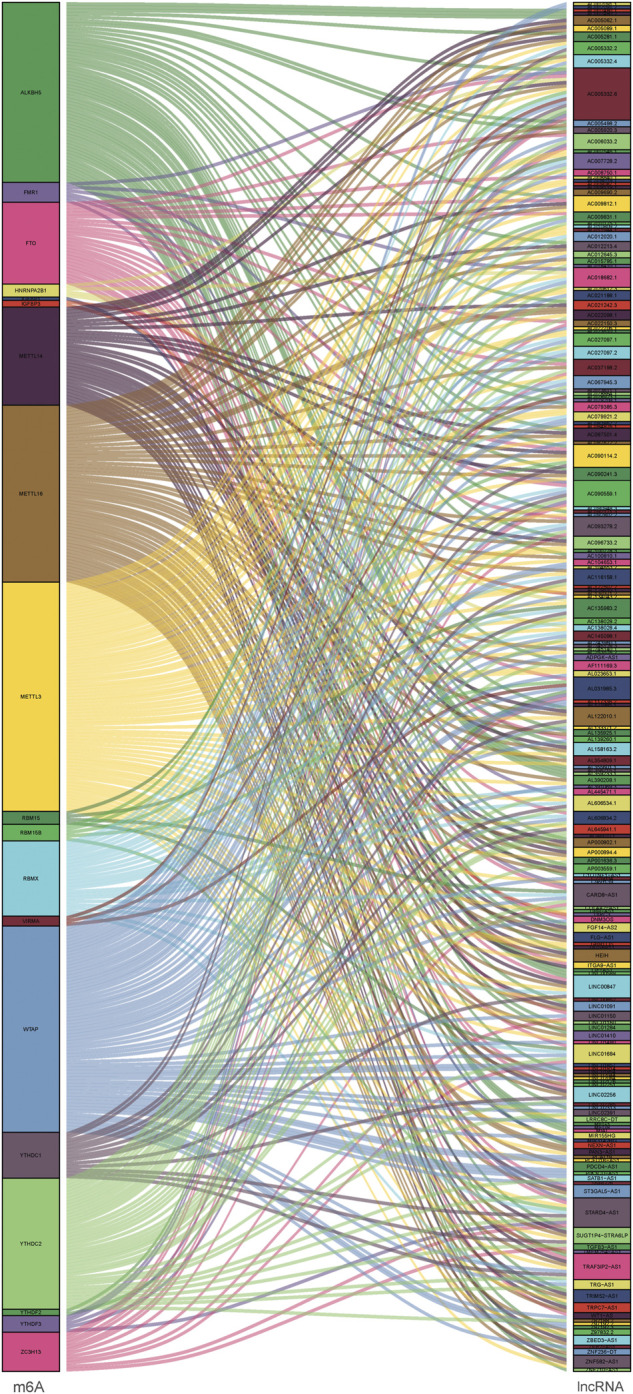
Co-expression network diagram of the 23 m6A modification regulators and m6A-related lncRNAs.

**FIGURE 3:**
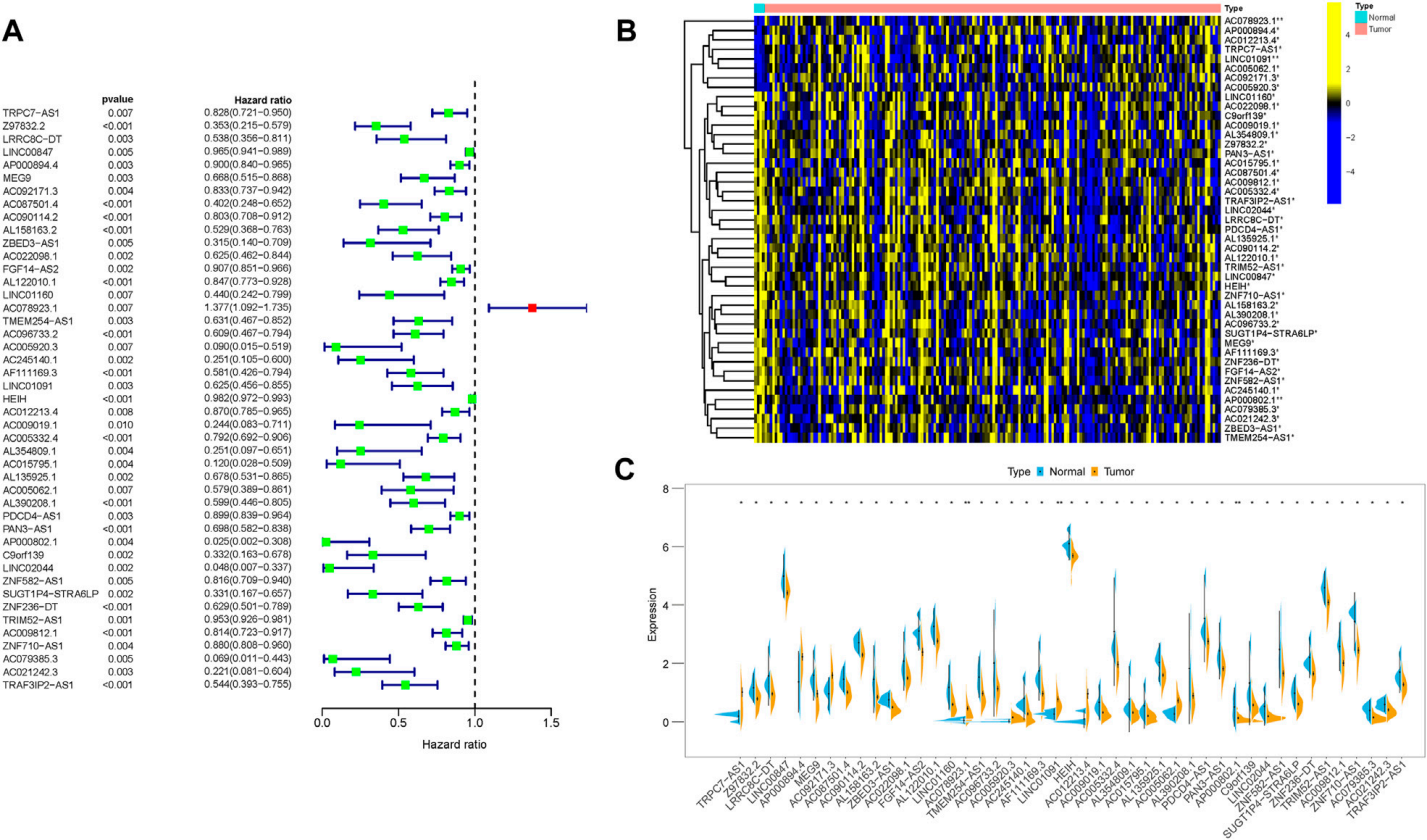
Prognostic value of m6A-related lncRNAs in PDAC. **(A)** Forest plot of prognostic m6A-related lncRNAs under univariable regression analysis. **(B)** Heatmap of m6A-related lncRNA expression levels in normal and tumor samples. **(C)** Differentially expression analysis of m6A-related lncRNAs in normal and tumor samples. *p < 0.05; **p < 0.01; ***p < 0.001.

Since lncRNAs were significantly associated with m6A RNA modification in the progression of tumor, lncRNAs and 23 m6A regulators coexpression network was assembled to visualize these m6A-related lncRNAs (Figure 2). In total, 173 lncRNAs were identified as m6A-related lncRNAs (*p* < 0.01 and | Pearson R| > 0.4) in this work (Supplementary Table S2). To further determine m6A-related lncRNAs with prognostic value, a univariate Cox regression analysis was performed. In total, 45 m6A-related lncRNAs significantly associated with OS time were determined, which was visualized in the forest plot (Figure 3A, Supplementary Table S3). The heatmap plot showed the expression distribution of these prognostic m6A-related lncRNAs (Figure 3B). In addition, expression levels of these prognostic m6A-related lncRNAs were significantly dysregulated in PDAC tumor samples (Figure 3C). Notably, stratification survival curves based on each m6A-related lncRNA indicated that abnormal expression levels of most m6A-related lncRNAs were significantly correlated with OS time (Supplementary Figure S3). These results highlighted indispensable functions of m6A-related lncRNAs in the development of PDAC.

### Development of m6A-related lncRNA clusters

**FIGURE 4:**
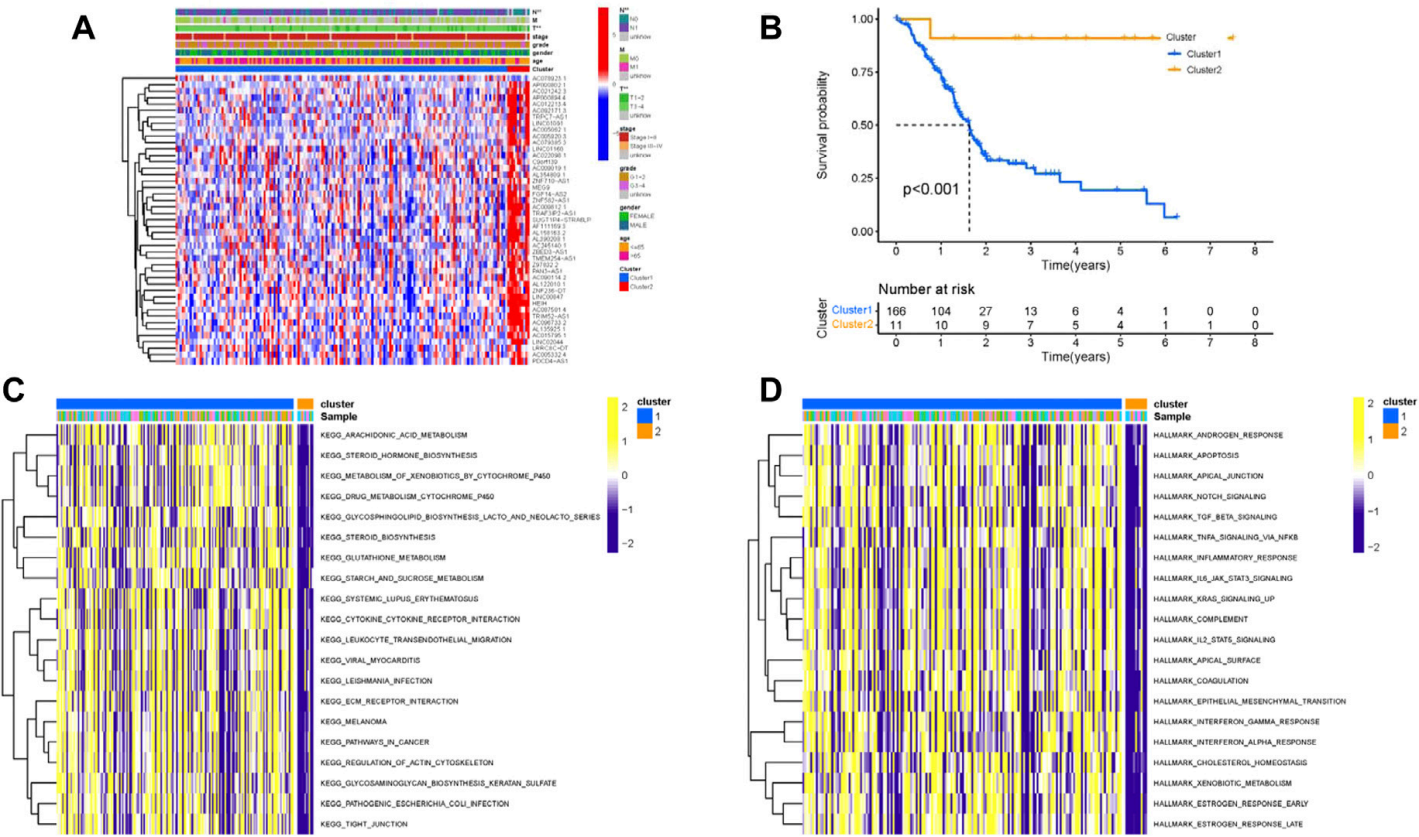
Clustering of prognostic m6A-related lncRNA expression profiling. **(A)** Unsupervised clustering of prognostic m6A-related lncRNAs to classify patients into different genomic subtypes, termed as m6A Cluster 1 and Cluster 2, respectively. The m6A clusters, age, gender, grade, tumor stage, and TNM status were used as patient annotations. **(B)** Survival curves of m6A clusters were estimated by the Kaplan–Meier plotter. (*p* < 0.001, log-rank test). GSVA enrichment analysis showed the activation states of biological pathways in distinct m6A clusters. The heatmap was used to visualize these biological processes; yellow represents activated pathways, and purple represents inhibited pathways. **(A)** KEGG; **(B)** Hallmarker.

The “ConsensusClusterPlus” R package was used to classify patients with distinct m6A-related lncRNAs patterns based on prognostic m6A-related lncRNAs (Supplementary Figures S4A–F). Thus, two different m6A-related lncRNAs patterns were finally determined using unsupervised clustering, including Cluster 1 (166 samples) and Cluster 2 (11 samples). The m6A-related lncRNAs expression distribution together with distinct m6A-related lncRNAs clusters and clinical traits are shown in Figure 4A. Kaplan–Meier survival analysis of two distinct m6A-related lncRNAs clusters indicated Cluster 2 experienced a prominent advantage in overall survival time compared with Cluster 1 (Figure 4B). To further elucidate the biological behaviors among two different m6A-related lncRNAs clusters, GSVA analysis was performed (Figures 4C and D and Supplementary Table S4). The results of GSVA presented that Cluster 1 was highly enriched in carcinogenic activation, including the Notch, TGF-β, IL2/STAT5, and IL6/JAK/STAT3 signaling pathways and epithelial–mesenchymal transformation.

### TEM characterization of m6A-related lncRNA clusters

**FIGURE 5:**
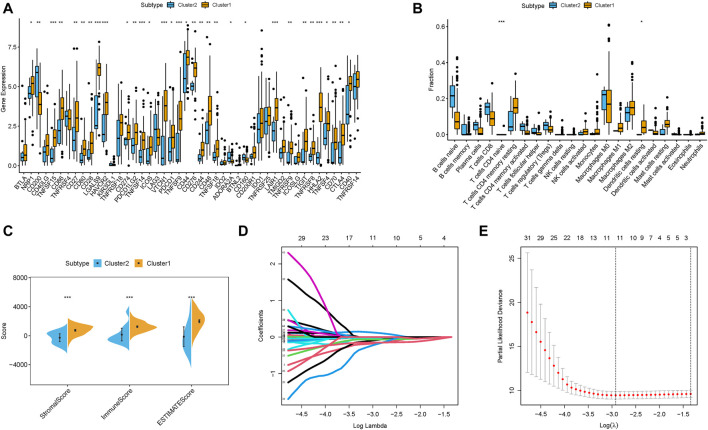
TIME contexture of m6A clusters in PDAC. **(A)** Differentially expression analysis of immune checkpoint blockade genes in m6A clusters. The fraction of tumor-infiltrating lymphocyte cells in m6A clusters. Within each group, the scattered dots represent TIME cell expression values. The thick line represents the median value. The bottom and top of the boxes are the 25th and 75th percentiles (interquartile range). The whiskers encompassed 1.5 times the interquartile range. The statistical difference of three gene clusters was compared through the Kruskal–Wallis H test. *p < 0.05; **p < 0.01; ***p < 0.001. **(B)** CIBERSORT algorithm; **(C)** ESTIMATE approach. Identification of the candidate stemness-related genes with prognostic value. **(D)** LASSO coefficient profiles of the expression of 45 candidate m6A-related lncRNAs. **(E)** Selection of the penalty parameter (λ) in the LASSO model *via* 10-fold cross-validation. The dotted vertical lines are plotted at the optimal values following the minimum criteria (left) and “one standard error” criteria (right).

First, 32 of 47 immunotherapy-related targets (CTLA‐4) were discovered to be significantly dysregulated between different risk groups (Figure 5A). Then, the TME characterized with the CIBERSORT algorithm was analyzed to compare the subpopulations of infiltrating immune cell abundances among two m6A-related lncRNAs Clusters (Figure 5B, Supplementary Table S5). Inactive immune cell subpopulations, such as resting dendritic cells and naive CD4 T cells, were markedly elevated in the m6A-related lncRNAs Cluster 1. Additionally, an analysis of immune infiltration with ESTIMATE was also further performed (Figure 5C, Supplementary Table S6). The results showed that Cluster 1 experienced a higher ESTIMATE score, immune score and stromal score relative to Cluster 2.

### Identification of the m6A-related lncRNA prognostic signature

Samples from TCGA-PAAD project were randomly assigned into training and testing subgroups. First, LASSO Cox regression analysis on 45 m6A-related lncRNAs correlated with prognosis was performed to construct risk model (Figure 5D). Finally, 11-lncRNAs (TRPC7-AS1, LRRC8C-DT, MEG9, AC087501.4, AC078923.1, AC245140.1, AC005332.4, PAN3-AS1, AP000802.1, ZNF236-DT, and ZNF710-AS1) prognostic risk model was established with the optimal value of λ (Figure 5E) to predict prognosis. The risk score was computed: risk score = (0.1155 ∗ AC078923.1 expression)–(0.0092 ∗ TRPC7-AS1 expression)–(0.1705 ∗ LRRC8C-DT expression)–(0.0323 ∗ MEG9 expression)–(0.0575 ∗ AC087501.4 expression)–(0.0832 ∗ AC245140.1 expression)–(0.0049 ∗ AC005332.4 expression)- (0.0660 ∗ PAN3-AS1 expression)–(0.1979 ∗ AP000802.1 expression)–(0.1527 ∗ ZNF236-DT expression)–(0.0038 ∗ ZNF710-AS1 expression). Then, PDAC samples of training group were partitioned into high-risk (*n* = 54) and low-risk subgroup (*n* = 54) based on the median value, and patients in testing group were assigned into low-risk (*n* = 37) and high-risk subgroup (*n* = 32) according to median value of training group.

### Prognostic value of the m6A-related lncRNA signature

**FIGURE 6:**
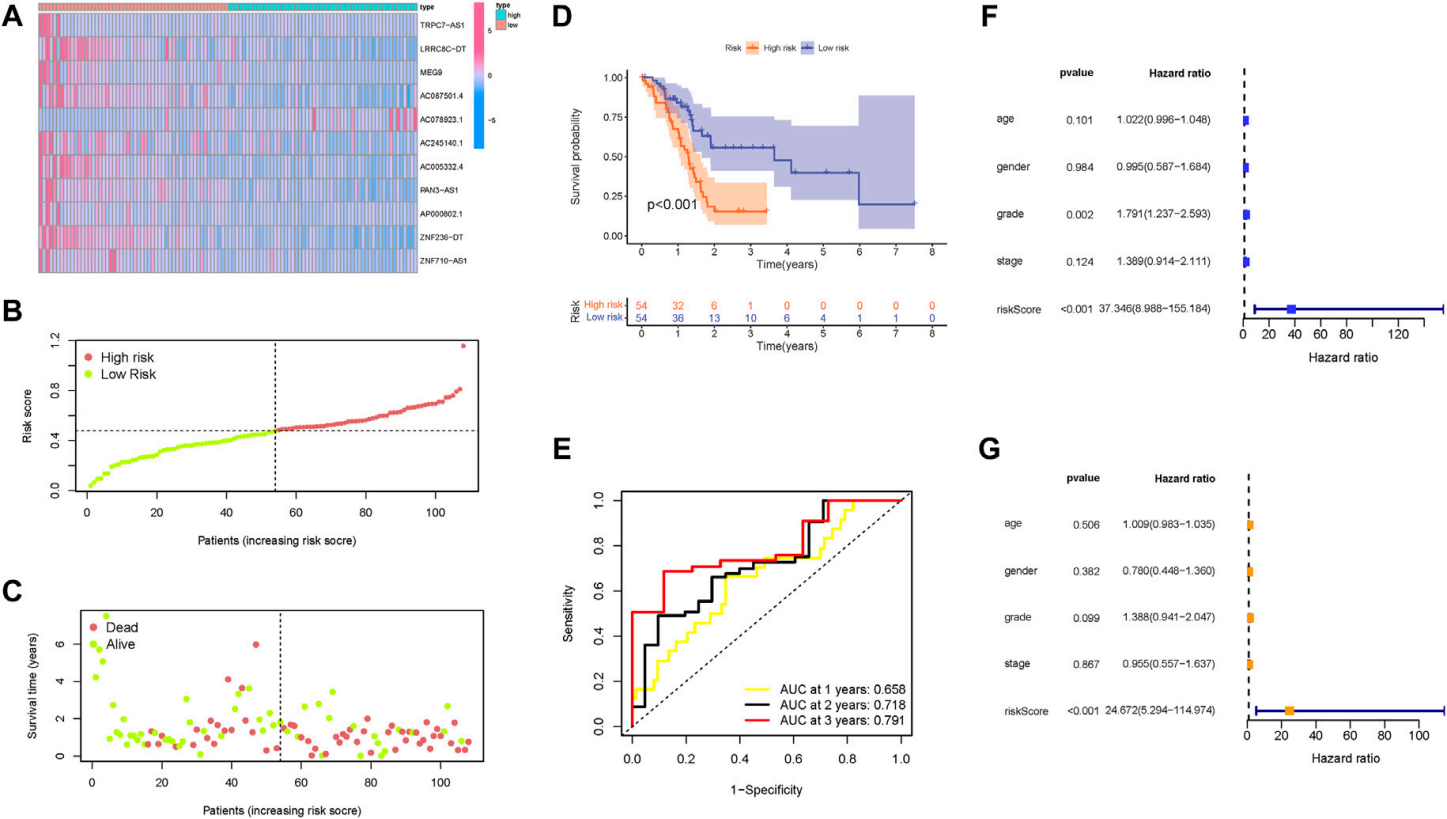
Confirmation of the prognostic value of the risk signature in the training group. **(A)** Heatmap presents the expression pattern of 11 hub m6A-related lncRNAs in each patient, where the colors yellow to blue represent alterations from high expression to low expression, respectively. **(B)** Distribution of the m6A-related lncRNA signature risk score. **(C)** Survival status and interval of PDAC patients. **(D)** Kaplan–Meier curve analysis presenting a difference in overall survival between the high-risk and low-risk groups. **(E)** Areas under curves (AUCs) of the risk scores for predicting 1-, 2-, and 3-year overall survival time. **(F)** Univariate Cox regression results of overall survival. The length of the horizontal line represents the 95% confidence interval for each group. The vertical dotted line represents the hazard ratio (HR) of all patients. **(G)** Multivariate Cox regression results of overall survival. The length of the horizontal line represents the 95% confidence interval for each group. The vertical dotted line represents the hazard ratio (HR) of all patients.

The distributions of lncRNAs expression values with corresponding risk groups are presented in Figure 6A. The allocations of risk score and dot pot of survival status highlighted that high-risk samples experienced significant shorter OS time (Figures 6B and C). Subsequently, the K–M survival curve indicated that patients with low risk exhibited a significantly better prognosis (*p* < 0.001; Figure 6D). The prognostic predictive value of risk score for OS was further validated by ROC curves, of which the area under the curve (AUC) was 0.658 at 1 year, 0.718 at 2 years, and 0.791 at 3 years (Figure 6E). Univariate and multivariate Cox regression were analyzed among the available variables to demonstrate the independent prognostic indicator of risk score. In single-factor regression analysis, the risk score was discovered to be significantly correlated with OS (HR = 37.346, 95% CI = 8.988–155.184, *p* < 0.001, Figure 6F). After correction for other confounding factors, the risk score still was an independent predictor for OS in the multivariate Cox regression analysis (HR = 24.672, 95% CI = 5.294–114.974, *p* < 0.001, Figure 6G).

### Validation of the m6A-related lncRNA signature

**FIGURE 7:**
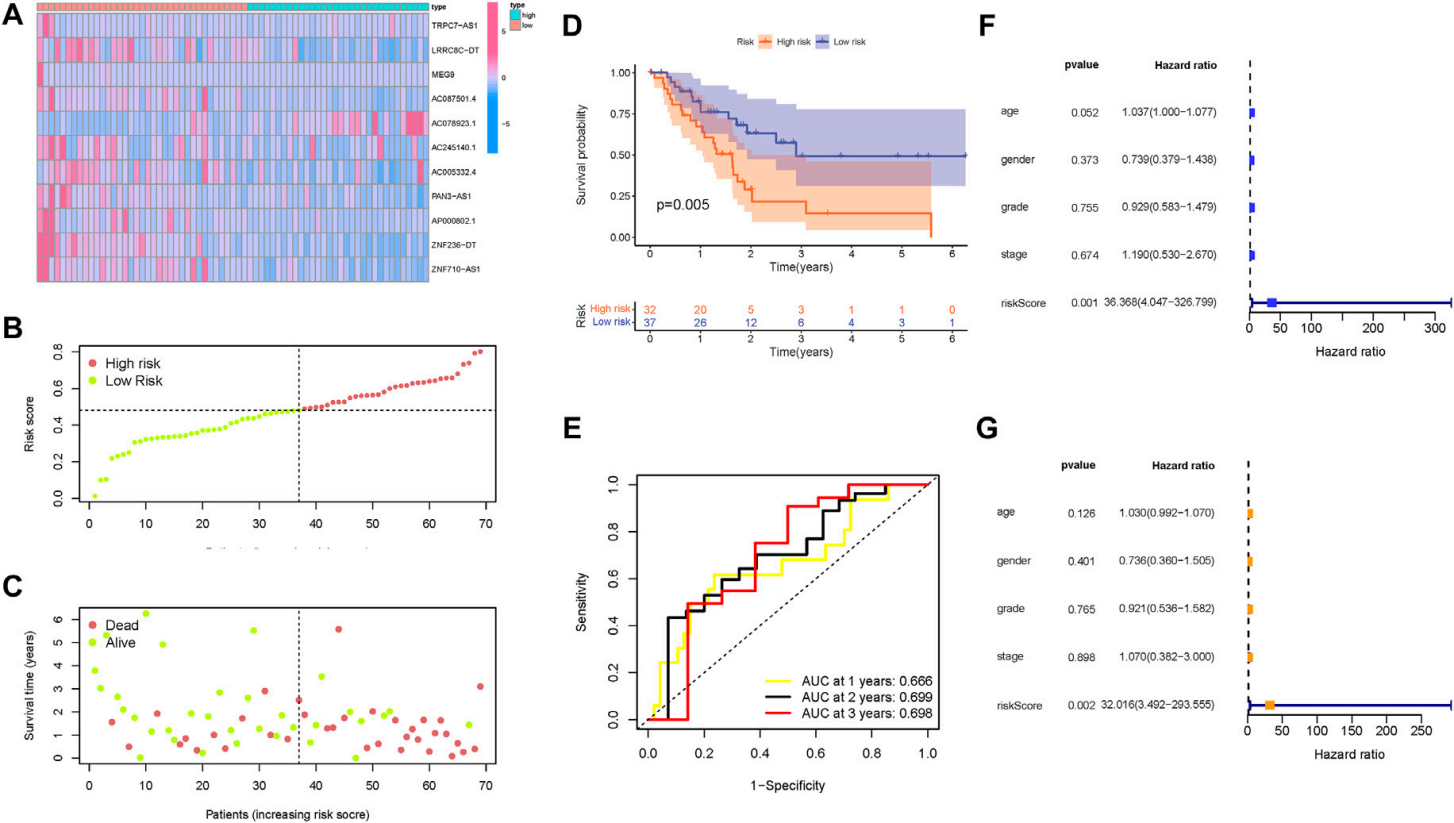
Validation of the prognostic value of the risk signature in the testing group. **(A)** Heatmap presents the expression pattern of 11 hub m6A-related lncRNAs in each patient, where the colors yellow to blue represented alterations from high expression to low expression, respectively. **(B)** Distribution of the m6A-related lncRNA signature risk score. **(C)** Survival status and interval of PDAC patients. **(D)** Kaplan–Meier curve analysis presenting a difference in overall survival between the high-risk and low-risk groups. **(E)** Areas under curves (AUCs) of the risk scores for predicting 1-, 2-, and 3-year overall survival time. **(F)** Univariate Cox regression results of overall survival. The length of the horizontal line represents the 95% confidence interval for each group. The vertical dotted line represents the hazard ratio (HR) of all patients. **(G)** Multivariate Cox regression results of overall survival. The length of the horizontal line represents the 95% confidence interval for each group. The vertical dotted line represents the hazard ratio (HR) of all patients.

Then, these findings were further validated in the testing group to confirm the prognostic significance of the risk model. The distribution of lncRNAs expression values, overall survival time, and risk score in the testing group are presented in Figures 7A–C, Figure 6C. Moreover, Kaplan–Meier curves revealed that samples in the low-risk group presented significantly longer OS time in the testing group (Figure 7D; *p* = 0.005). The AUC values of ROC curves reached up to 0.65 in the testing group (Figure 7E), indicating the outstanding predictive performance of the risk model. Consistent with the findings derived from the training group, the risk model was an independent predictive factor in both univariable and multivariable COX regression analysis of the testing group (Figures 7F and G).

### Clinical significance of the m6A-related lncRNA signature

**FIGURE 8:**
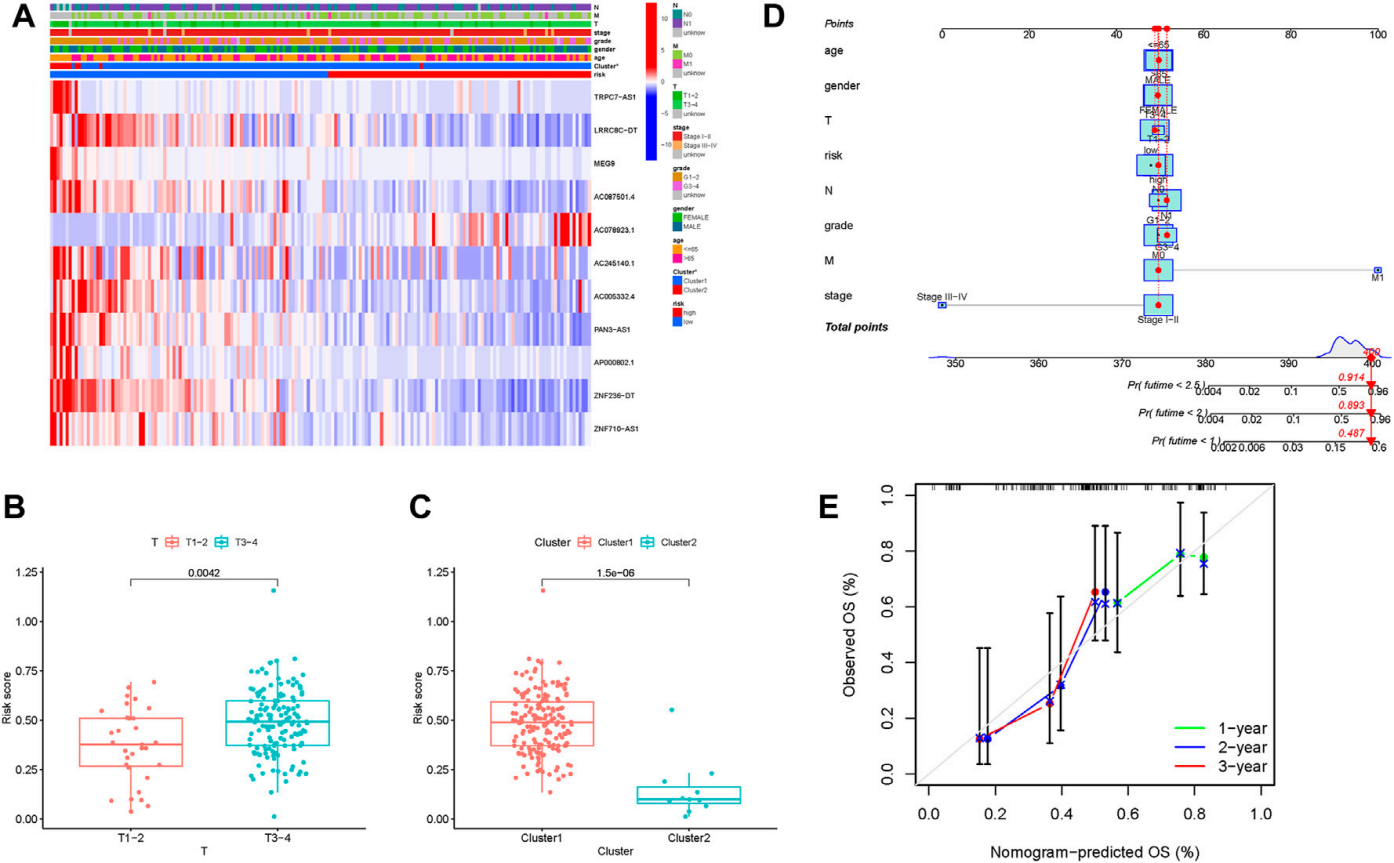
Correlation analysis of the risk score with clinical factors, m6A clusters. **(A)** Heatmap of m6A-related lncRNAs together with clinical factors and m6A clusters. Blue represents downregulated expression, and red represents upregulated expression. **(B)** Difference in the risk score between T1-2 and T3-4 subgroups. **(C)** Difference in the risk score between m6A Cluster 1 and m6A Cluster 2. **(D)** Nomogram was assembled by clinical traits and risk signatures for predicting the survival of PDAC patients. **(E)** Nomogram calibration curves for 1-, 2-, and 3-year overall survival time.

First, the distribution of m6A-related lncRNAs expression with clinical variables in different risk groups was investigated and visualized (Figure 8A). For T1-2 samples and T3-4 samples, the risk score exhibited a higher trend in T3-4 samples (Figure 8B). It was discovered that patients from Cluster 1 also exhibited a significant increase in the risk score (Figure 8C). These findings highlighted that m6A-related lncRNAs risk score was significantly correlated with clinicopathological features.

### Establishment of the prognostic nomogram

To demonstrate the best prognostic predictive factor of risk score, gender, age, tumor grade, clinical staging, and TNM status were used as the candidate prognostic predictors. These predictive variables were adopted into the ROC analysis for 1-, 2-, and 3-year OS time, and the risk score was discovered to reach the highest AUC values (Supplementary Figures S5A–C). Subsequently, a prognostic nomogram, including clinical traits and risk score, was established to predict the overall survival rate in a quantitative manner (Figure 8D). In addition, calibrate curves were analyzed to demonstrate the great sensitivity and specialty of the as-constructed nomogram (Figure 8E).

### Enrichment of signaling pathways in low-/high-risk groups

**FIGURE 9:**
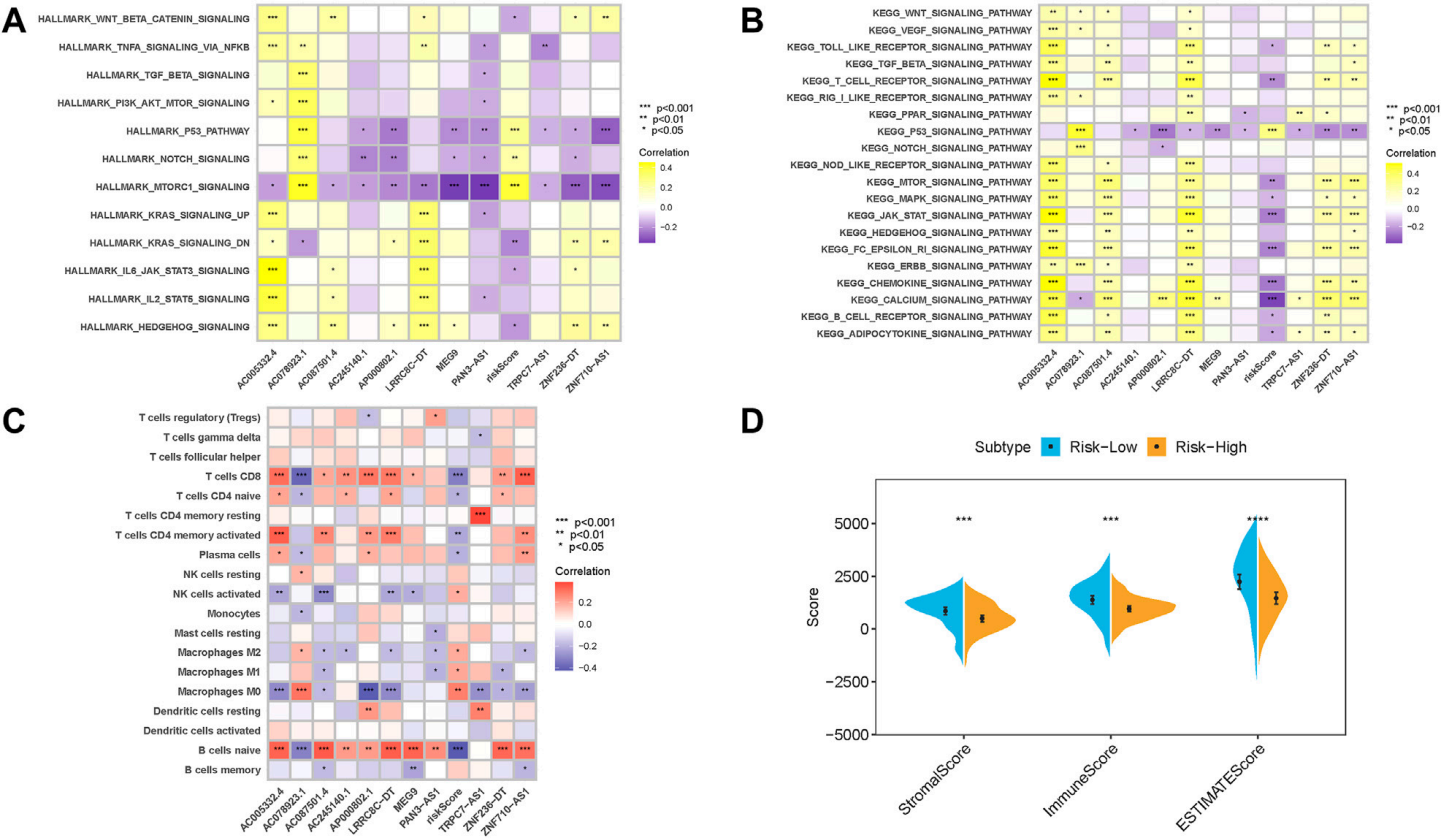
Enrichment pathways of GSVA. **(A)** Heatmap showing the correlation of representative pathway terms of Hallmark with the risk score. **(B)** Heatmap showing the correlation of representative pathway terms of KEGG with the risk score. **(C)** Correlation analysis of the risk score with immune infiltration. *p < 0.05; **p < 0.01; ***p < 0.001. **(C)** CIBERSORT algorithm; **(D)** ESTIMATE approach.

To further reveal the biological roles of distinct risk groups in tumorigenicity and progression, gene set variation analysis (GSVA) was performed (Figures 9A and B). Samples from the low-risk group showed heightened activities of the ERBB, MAPK, KARS, IL6/JAK/STAT3, and Wnt-β-catenin signaling pathways. Most genes with high expression levels in the high-risk group were enriched in the mTORC1, P53, and Notch signaling pathways.

### Correlation of the risk signature with TME features

Since m6A-related lncRNAs risk score and infiltration immune cells shared intrinsic interaction, the potential contribution of the risk score in the characterization of TME was further investigated. These results revealed that the risk score was negatively and significantly associated with subpopulations of CD8^+^ T cells, activated CD4 memory T cells, and plasma cells, while it was positively associated with the abundance of M0 and M2 macrophages (Figure 9C). Moreover, the results of ESTIMATE analysis exhibited that stromal score, immune score, and ESTIMATE score experienced a significantly higher trend in the low-risk group (Figure 9D).

### Prediction of immunotherapeutic outcomes

**FIGURE 10:**
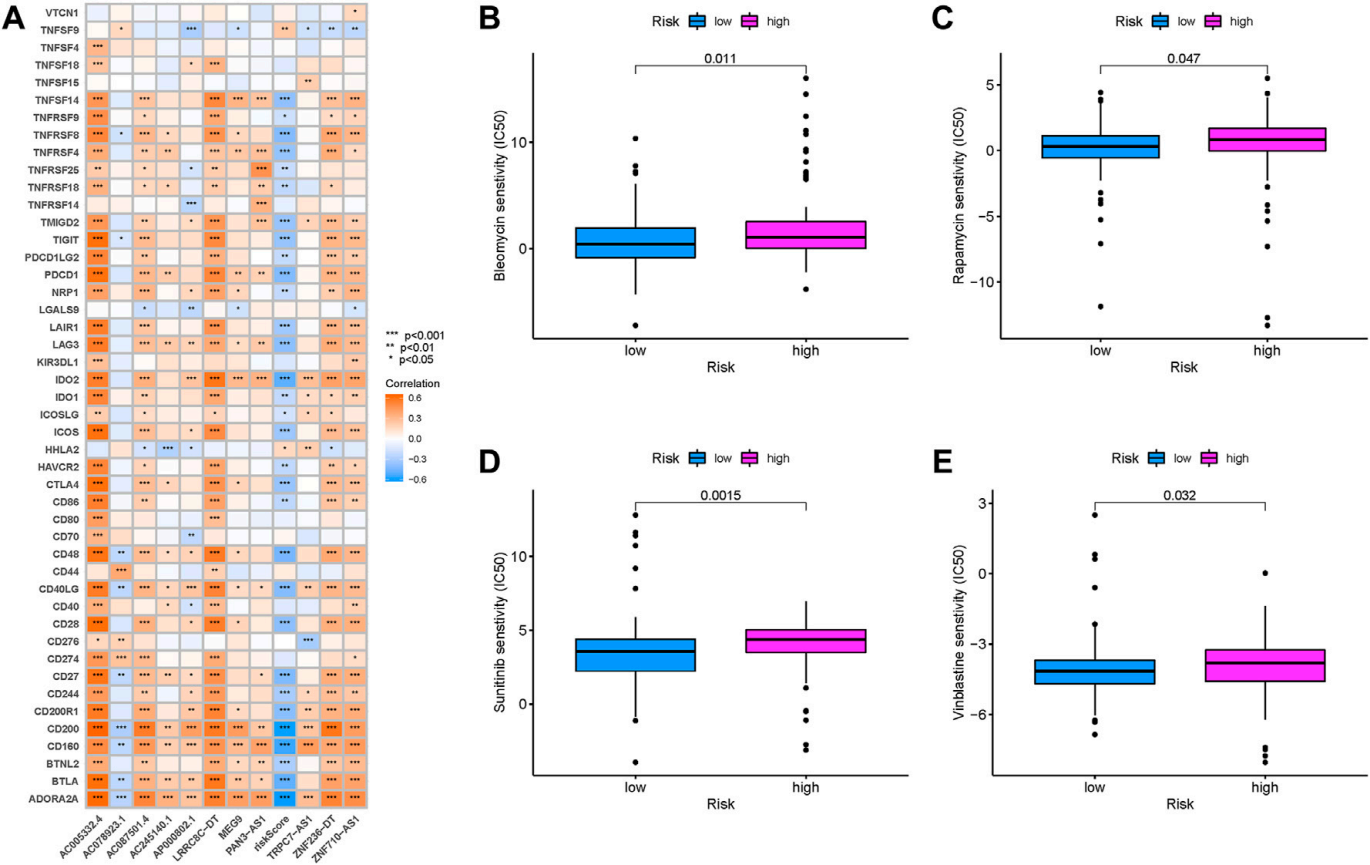
Prediction of immunotherapeutic response. **(A)** Correlation of expression level of immune checkpoint blockade genes with the risk score. Estimation of the risk score in chemotherapeutic effect. **(B)** Sensitivity analysis of bleomycin in patients with high- and low-risk scores. **(C)** Sensitivity analysis of rapamycin in patients with high- and low-risk scores. **(D)** Sensitivity analysis of sunitinib in patients with high- and low-risk scores. **(E)** Sensitivity analysis of vinblastine in patients with high- and low-risk scores.

Since the immunotherapeutic information was not available in TCGA-PAAD project, the correlation of risk score response to immunotherapy was not able to explore. For that, a total of immune checkpoint blockade-related genes (PDCD1 and CTLA4) were detected in different risk groups, and it was discovered that most ICB-related genes experienced a negative correlation with risk score (Figure 10A), suggesting that risk score was correlated with the response to immunotherapies, further predicting prognosis accordingly.

### Prediction of response to chemotherapy

Based on the pRRophetic algorithm, the IC_50_ of four chemotherapeutic drugs (bleomycin, rapamycin, sunitinib, and vinblastine) were estimated in PDAC patients. Bleomycin, rapamycin, sunitinib, and vinblastine exhibited higher IC_50_ in patients with high-risk scores (all *p* < 0.05; Figures 10B–E). These results supported the suggestion of patients with higher risk scores were sensitive to chemotherapeutic drugs.

### The potential role of TRPC7-AS1 in prognosis and pathway enrichment

**FIGURE 11:**
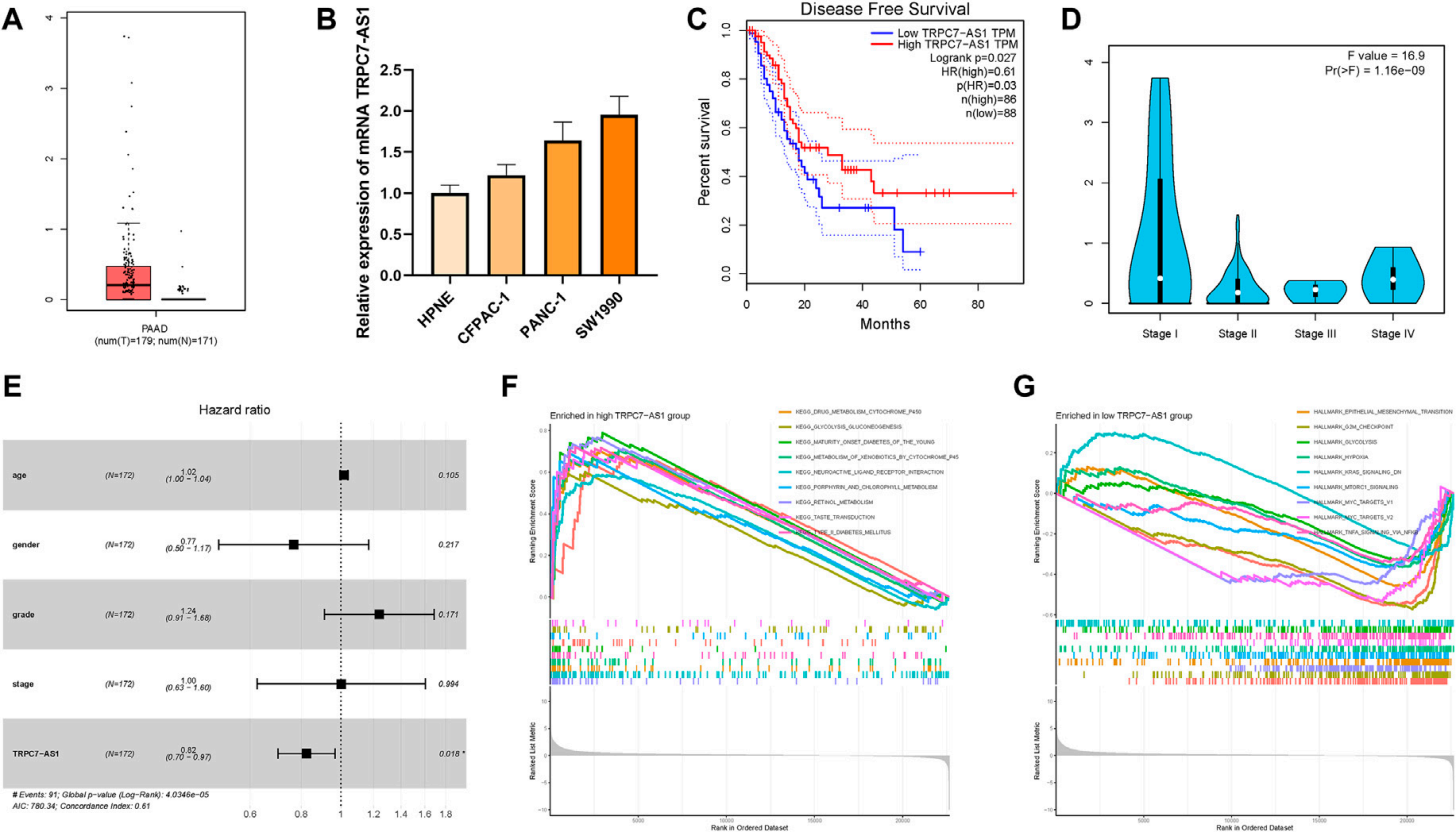
Clinical significance of TRPC7-AS1 in PDAC. TRPC7-AS1 are upregulated in PDAC samples based on TCGA and GTEx datasets **(A)** and cell lines **(B)**, and higher TRPC7-AS1 expression level was significantly correlated with improved prognosis **(C)**. **(D)** Expression of TRPC7-AS1 had a significant difference between major pathological stages. **(E)** Multivariate Cox regression results of overall survival. The length of the horizontal line represents the 95% confidence interval for each group. The vertical dotted line represents the hazard ratio (HR) of all patients. **(F)** Enriched gene sets in KEGG collection by the high TRPC7-AS1 expression sample. **(G)** Enriched gene sets in Hallmarker collection by the low TRPC7-AS1 expression sample.

TRPC7-AS1 was the hub lncRNA with the most significant dysregulated expression level among these prognostic m6A-related lncRNAs. For that, the biological function of TRPC7-AS1 in PDAC was further investigated in subsequent analyses. The expression levels of TRPC7-AS1 were between tumor samples and normal tissues according to TCGA and GTEx datasets. For tumor tissues and normal specimens, the TRPC7-AS1 expression value exhibited a higher trend in tumor tissues (Figure 11A). With the help of qRT-PCR, the expression levels of TRPC7-AS1 in human pancreatic cell lines and four distinct pancreatic cancer cell lines were detected. Consistently, normal pancreatic cells presented significantly lower TRPC7-AS1 values than PDAC cells (Figure 11B). To estimate the prognostic performance of TRPC7-AS1, survival analysis was performed between TRPC7-AS1 low- and high-expressed samples. It was discovered that a higher expression level of TRPC7-AS1 significantly suggested a higher DFS rate (*p* = 0.00053, Figure 11C). The expression level analysis among major clinical stages showed that TRPC7-AS1 expressed significantly different among distinct clinicopathological stages (Figure 11D, F = 16.9 and *p* = 1.16e-09). In multivariate regression analysis, TRPC7-AS1 was discovered to be significantly correlated with OS (HR = 0.82, 95% CI = 0.70–0.97, *p* = 0.018, Figure 11E). According to the median expression of TRPC7-AS1, all samples were divided into a high expression group and a low expression group. Then, GSEA was performed to identify the functional enrichment of high and low TRPC7-AS1 gene expression. KEGG enrichment term exhibited that high expression of TRPC7-AS1 was mainly associated with drug metabolism cytochrome P450 and metabolism of heterologous substances by cytochrome P450 (Figure 11F). Gene sets, including epithelial–mesenchymal transformation, glycolysis, and hypoxia were enriched in patients with low TRPC7-AS1 expression (Figure 11G).

## Discussion

An increasing number of studies have highlighted that m6A modification and lncRNAs served as a nonnegligible regulator in antitumor effects, inflammation, and innate immunity (Fang and Fullwood, 2016; Chen et al., 2019; Shulman and Stern-Ginossar, 2020; Wu et al., 2020; Xu et al., 2021a). As plenty of research studies concentrated on single lncRNA or several m6A regulators, the comprehensive analysis mediated by integrated m6A RNA modification regulators and lncRNAs has not been elucidated in PDAC. Determination of the biological function of m6A-related lncRNA patterns in subtypes identification, prognostic prediction, TME characterization, enrichment assignment, and therapeutic estimation directs a crucial approach for our understanding of the antitumor molecular mechanism for further optimizing precision therapeutic interventions.

In this work, m6A-related lncRNAs were determined using univariate Cox regression, followed by Pearson correlation. Then, two different m6A-related lncRNAs patterns associated with distinct overall survival were identified, which were characterized by diverse anticancer immunity and signaling pathways. Notably, Notch, TGF-β, IL2/STAT5, and IL6/JAK/STAT3 signaling pathways and epithelial–mesenchymal transformation were activated in Cluster 1. These results showed that the underlying molecular mechanism is diverse well between different clusters.

Subsequently, LASSO algorithm analysis was used to construct the risk model, and the final 11 significant m6A-related lncRNAs were assigned to the prognostic risk model. Its prognostic value was validated by the Kaplan–Meier survival analysis, the time-dependent ROC analysis, and the univariate/multivariate Cox regression model. Further validation was performed in the testing group. Then, the correlation of risk score with clinical features was explored, and a novel prognostic clinical-risk nomogram was constructed and confirmed to quantify the overall survival rate in individual samples.

Since m6A-related lncRNAs were associated with TME features, the potential role of risk score was further investigated to reveal the TME diversity. The results of immune infiltrating highlighted that risk score experienced a negative correlation with an abundance of activated immune cells (CD8^+^ T cells, activated CD4 memory T cells), while it was positively associated with immunosuppressive cells (M2 macrophages). Interestingly, the low-risk group was characterized by a higher stromal score and immune score. Taken together, the low-risk group was characterized by the presence of infiltrating immune cells and stromal elements, which could be considered an immune-excluded phenotype. Although high infiltration of immune cells presented in the immune-excluded phenotype, these immune cells, the penetration of which into the parenchyma of the tumor was impeded by the abundant stromal element, were unable to function as recognition and elimination of cancer cells. On the contrary, the high-risk group was characterized by the absence of infiltrating immune cells and weakened immune activity, which was regarded as an immune-desert phenotype. Based on the TME characteristics in each risk group, it supported the robustness of risk scoring of immune phenotypes with distinct m6A-related lncRNA patterns.

In the absence of an appropriate immunotherapy-based PDAC dataset, immunotherapeutic hub targets were integrated to confirm the predictive validity of the risk score. Our findings indicated that the risk score was negatively correlated with ICB-related gene expression levels (CD274). It suggested that patients with a lower risk score, corresponding to the immune-excluded phenotype, might be more suitable for ICB immunotherapy mainly because of more influenced by immune checkpoint blockade. These results supported that the m6A-related lncRNAs scoring scheme could contribute to the identification of immune phenotypes and optimization of immunotherapeutic practice.

It is worth mentioning that GSVA results indicated that ERBB, MAPK, KARS, Wnt-β-catenin, and IL6/JAK/STAT3 signaling pathways were significantly activated in samples with low-risk, whereas high-risk group were associated with the mTORC1, P53, and Notch signaling pathways. In addition, the risk scoring scheme revealed that sensitivity to chemotherapy drugs was associated with risk scores. For that, PDAC patients might be more suitable for distinct combination administration with molecule-targeting and chemotherapeutic agents according to risk stratification.

Among these m6A-related lncRNAs in the risk model, the biological functions of TRPC7-AS1 have not been revealed yet in PDAC. In addition, TRPC7-AS1 expression was discovered to independently affect the prognosis of patients with PDAC. Transient receptor potential (TRP) ion channel is a transmembrane protein, especially TRPC7-AS1, which plays key roles in pain, mechanical injury, osmotic pressure perception, and temperature perception by changing intracellular calcium concentration or cell membrane potential (Moran, 2018). Recently, several research studies focusing on the biological roles of TRPC7-AS1 have been published. Zhu, S et al. reported that TRPC7-AS1 could be a potential therapeutic target or diagnostic marker for HCC (Zhu et al., 2021). Qi T et al. demonstrated that LncRNA TRPC7-AS1 relieves miR-4769–5p-induced inhibition on HPN *via* acting as a ceRNA, thus, regulating NPC viability, senescence, and ECM synthesis (Wang et al., 2020). In this work, prognostic performance and effects on clinical outcome and mechanism of TRPC7-AS1 were elucidated. It was discovered that TRPC7-AS1 is significantly overexpressed in PDAC cells and could play as a poor prognostic predictor in PDAC. Notably, TRPC7-AS1 was demonstrated to be an independent prognostic factor in PDAC. In addition, TRPC7-AS1 experienced a significant negative correlation with epithelial–mesenchymal transformation, glycolysis, and hypoxia in PDAC. However, the underlying biomolecular mechanism of TRPC7-AS1 in PDAC remains obscure, requiring further validation.

In this work, diverse m6A-related lncRNA patterns among 177 PDAC samples based on 45 prognostic m6A-related lncRNAs were comprehensively identified. In addition, subtype clustering based on m6A-related lncRNAs was constructed to quantify the m6A-related lncRNA patterns. Finally, the m6A-related lncRNA scoring scheme was established to reveal prognostic prediction, underlying signaling pathways, TEM features, and chemotherapeutic prediction. Finally, the potential role of TRPC7-AS1 was explored in PDAC. Collectively, the comprehensive evaluation of m6A-related lncRNA patterns in PDAC will provide novel insights into molecular mechanisms and therapeutic strategies.

## Data Availability

The datasets presented in this study can be found in online repositories. The names of the repository/repositories and accession number(s) can be found in the article/Supplementary Material.

## Glossary

CD274: also known as PD-L1
CI: confidence interval
CNV: copy number variation
CTLA‐4: cytotoxic T‐lymphocyte antigen 4
DEL: deletion
DFS: disease-free survival
FDR: false discovery rate
FPKM: fragments per kilobase of exon model per million mapped fragments
GDC: Genomic Data Commons
GDSC: Genomics of Drug Sensitivity in Cancer
GSVA: gene set variation analysis
HR: hazard ratio
IC_50_: half-maximal inhibitory concentration
ICB: immune checkpoint blockade
IDO1: indoleamine 2,3‐dioxygenase 1
KEGG: Kyoto Encyclopedia of Genes and Genomes
K–M: Kaplan–Meier
K–W: Kruskal–Wallis
lncRNA: long noncoding RNA
MDS: multidimensional scaling
m6A: methylation of N6 adenosine
OS: overall survival
PCA: principal component analysis
PDAC: pancreatic ductal adenocarcinoma
PDCD1: also known as PD-1
PDCD1LG2: also known as PD‐L2
ROC: receiver operating characteristic
SNV: simple nucleotide variation
TCGA: The Cancer Genome Atlas
TICs: tumor‐infiltrating immune cells
TILs: tumor-infiltrating lymphocytes
TIM‐3: T‐cell immunoglobulin domain and mucin domain‐containing molecule‐3
TME: tumor microenvironment
TNM: tumor node metastasis
TPM: transcripts per kilobase million
