# Supplementary material for: Prognostic N6-methyladenosine (m6A)-related lncRNA patterns to aid therapy in pancreatic ductal adenocarcinoma
Source: Front Genet. 2022 Sep 26;13:866340. doi: 10.3389/fgene.2022.866340 (PMC9549010; doi:10.3389/fgene.2022.866340)
Supplement: Supplementary file 1 [file DataSheet2.pdf]

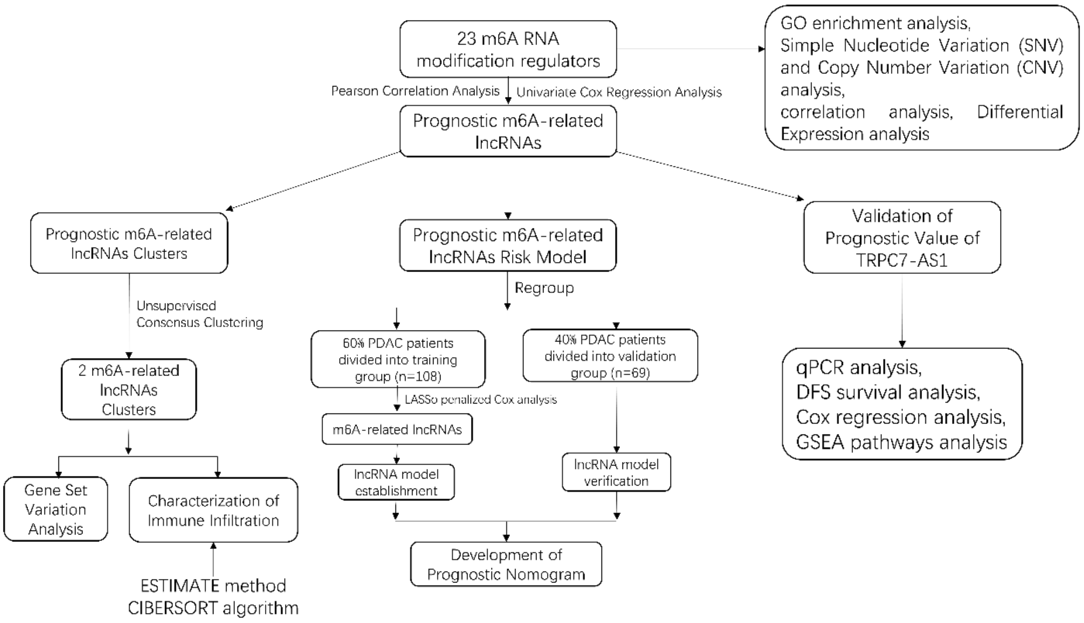

Figure S1. Overall research design. Flow-process diagram presenting the process of comprehensive analysis.

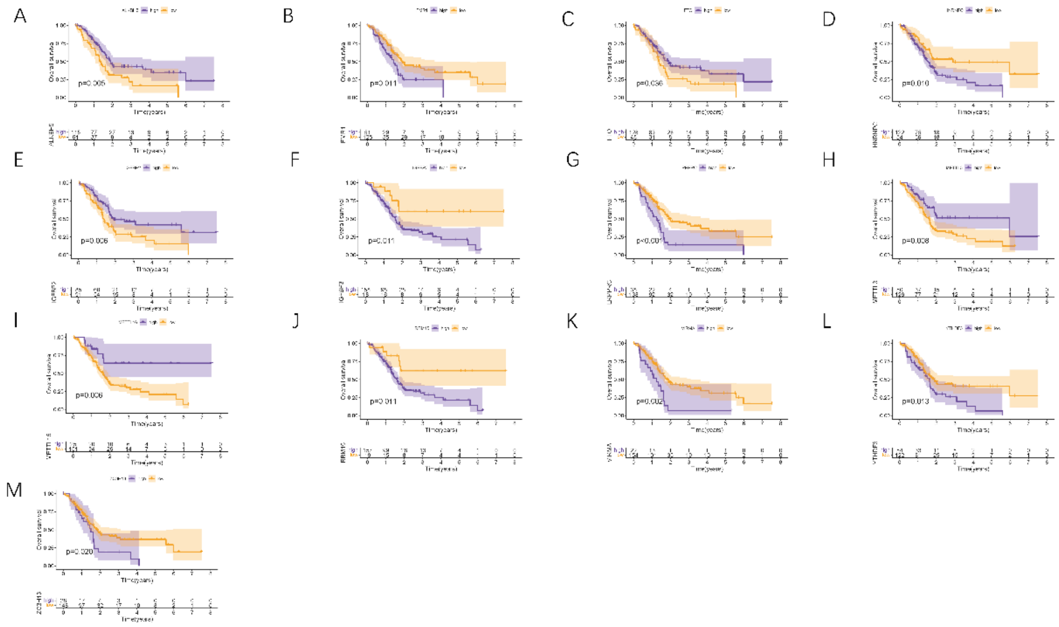

Figure S2. Survival analysis between high- and low- expression groups of hub m6A genes. (A) ALKBH5, (B) FMR1, (C) FTO, (D) HNRNPC, (E) IGFBP2, (F) IGFBP3, (G) LRPPRC, (H) METTL3, (I) METTL16, (J) RBM15, (K) VIRMA, (L) YTHDF3, and (M) ZC3H13.

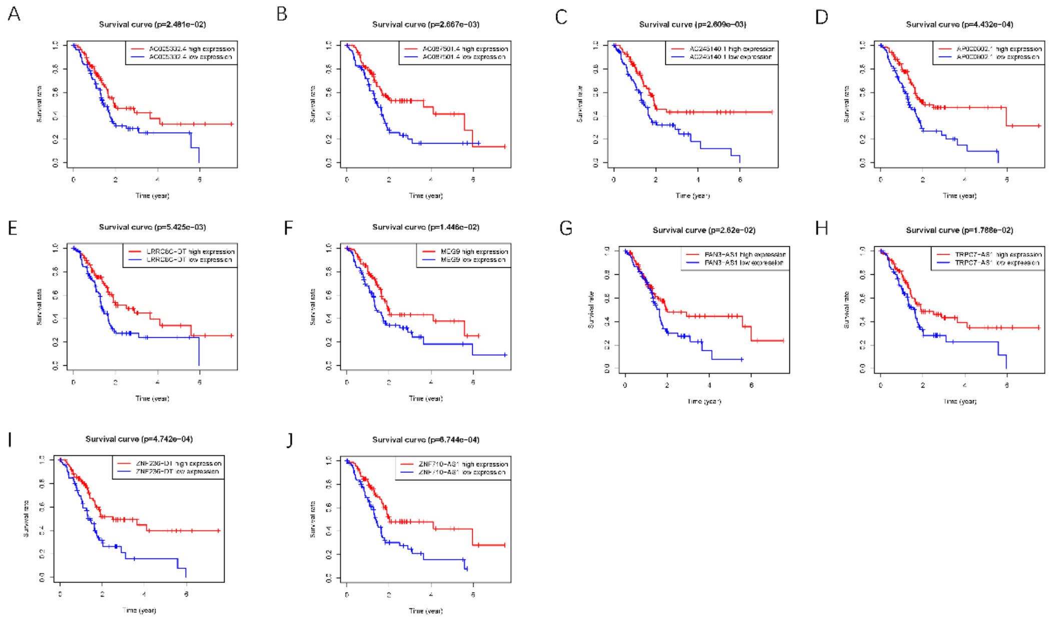

Figure S3. Survival analysis between high- and low- expression groups of hub m6A-related lncRNAs. (A) AC005332.4, (B) AC087501.4, (C) AC245140.1, (D) AP000802.1, (E) LRRC8C-DT, (F) MEG9, (G) PAN3-AS1, (H) TRPC7-AS1, (I) ZNF236-DT, and (K) ZNF710-AS1.

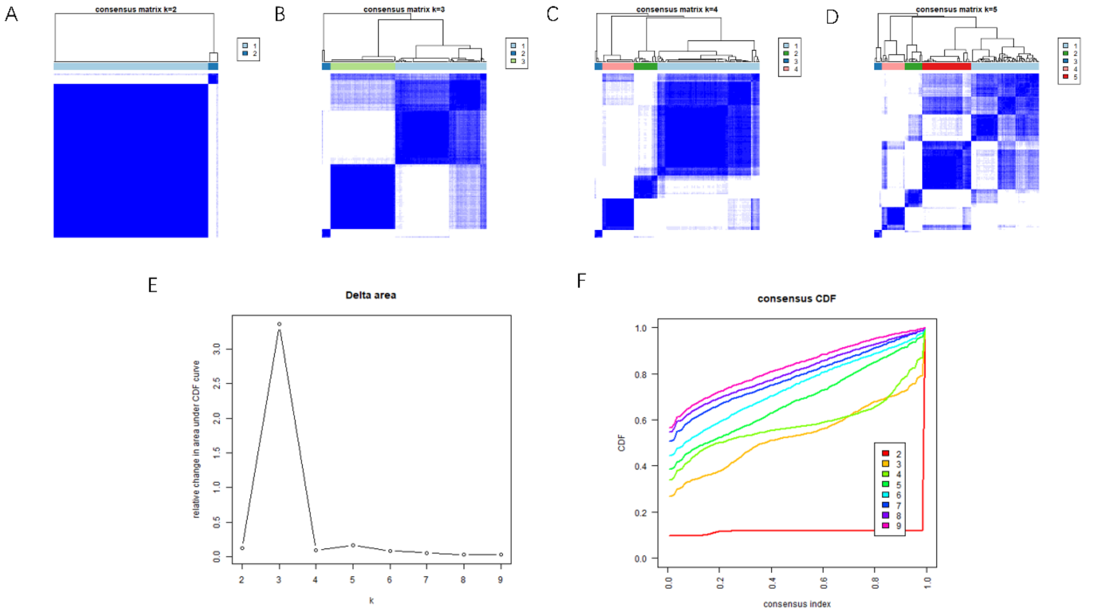

Figure S4. Consensus clustering based on prognostic m6A-related lncRNAs. (A-D) Consensus matrixes of HCC samples for each k (k = 2–5), displaying the clustering stability using 1000 iterations of hierarchical clustering. (E) The relative change in area under the CDF curve for k = 2–9. (F) Area under cumulative distribution function (CDF) curve when index k ranges from 2 to 10.

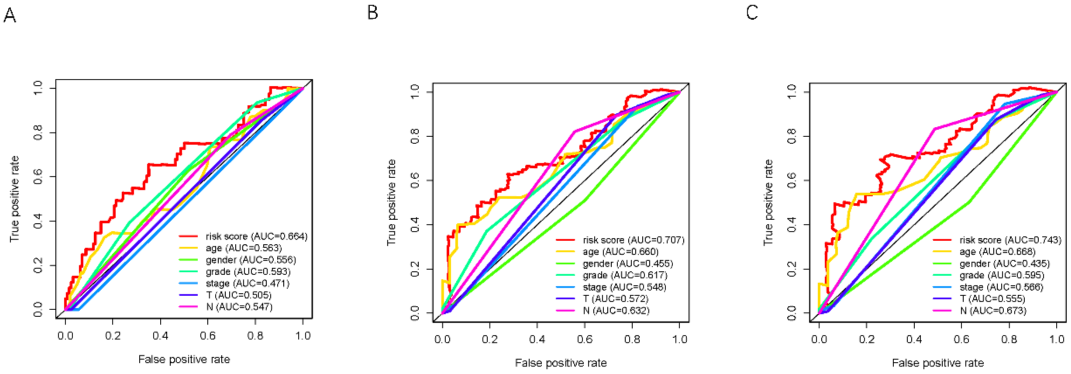

Figure S5. (A) Areas under curves (AUCs) of the risk scores for predicting 1-year overall survival time with other clinical characteristics. (B) Areas under curves (AUCs) of the risk scores for predicting 2-year overall survival time with other clinical characteristics. (C) Areas under curves (AUCs) of the risk scores for predicting 3-year overall survival time with other clinical characteristics.
